# Risk of transfusion-transmitted malaria: evaluation of commercial ELISA kits for the detection of anti-*Plasmodium* antibodies in candidate blood donors

**DOI:** 10.1186/s12936-019-2650-0

**Published:** 2019-01-22

**Authors:** Valentina D. Mangano, Francesca Perandin, Natalia Tiberti, Massimo Guerriero, Franca Migliaccio, Marco Prato, Lucia Bargagna, Stefano Tais, Monica Degani, Federica Verra, Zeno Bisoffi, Fabrizio Bruschi

**Affiliations:** 1 Department of Translational Science, University of Pisa, Pisa, Italy; 2 Department of Laboratory Medicine, Pisa University Hospital, Pisa, Italy; 3 Centre for Tropical Diseases, IRCCS Sacro Cuore-Don Calabria Hospital, Negrar, Verona Italy; 4 Department of Computer Science, University of Verona, Verona, Italy; 5 Department of Diagnostic and Public Health, University of Verona, Policlinico “G. B. Rossi”, Verona, Italy

**Keywords:** *Plasmodium*, Transfusion transmitted malaria, ELISA, IFAT

## Abstract

**Background:**

Transfusion with *Plasmodium*-infected blood represents a risk for malaria transmission, a rare but severe event. Several non-endemic countries implement a strategy for the screening of candidate blood donors including questionnaire for the identification of at-risk subjects and laboratory testing of blood samples, often serology-based, with temporary deferral from donation for individuals with a positive result. In Italy, the most recent legislation, issued in November 2015, introduced the use of serological tests for the detection of anti-*Plasmodium* antibodies.

**Methods:**

In the absence of a gold standard for malaria serology, the aim of this work was to evaluate five commercial ELISA kits, and to determine their accuracy (sensitivity and specificity) in comparison to immuno-fluorescence antibody test (IFAT), and their agreement (concordance of results). Serum samples from malaria patients or from subjects with malaria history (N = 64), malaria naïve patients with other parasitic infections (N = 15), malaria naïve blood donors (N = 8) and malaria exposed candidate blood donors (N = 36) were tested.

**Results:**

The specificity of all ELISA kits was 100%, while sensitivity ranged between 53 and 64% when compared to IFAT on malaria patients samples. When tested on candidate blood donors’ samples, ELISA kits showed highly variable agreement (42–94%) raising the possibility that the same individual could be included or excluded from donation depending on the test in use by the transfusion centre.

**Conclusions:**

These preliminary results indicate how the lack of a gold standard for malaria serology must be taken into account in the application and future revision of current legislation. There is need of developing more sensitive serological assays. Moreover, the adoption of a unique serological test at national level is recommended, as well as the development of screening algorithms based on multiple laboratory tests, including molecular assays.

**Electronic supplementary material:**

The online version of this article (10.1186/s12936-019-2650-0) contains supplementary material, which is available to authorized users.

## Background

Transfusion-transmitted malaria (TTM) is an alternative accidental route of malaria infection caused by the transfusion of whole blood, or a blood component, from an infected donor harbouring *Plasmodium* parasites to a recipient. The infection may be responsible for the development of severe clinical symptoms in the recipients, especially in malaria naïve individuals, and may become life threatening [1, 2].

Six species of *Plasmodium* are currently known to cause malaria in humans with 216 million new cases and 445,000 deaths estimated in 2016 [3]. *Plasmodium falciparum,* the most diffused species—especially in sub-Saharan Africa (SSA)—is responsible for the vast majority of severe and fatal cases. *Plasmodium vivax*, mainly present in Central and South America and Southeast Asia can also cause severe disease, although the fatality rate is lower compared to *P. falciparum* [4]. Interestingly, *P. vivax* and *Plasmodium ovale* (the latter being mainly observed in SSA) have a dormant liver stage—hypnozoite—which can relapse months or years after treatment completion; while the worldwide distributed *Plasmodium malariae* infection can persist indefinitely [5, 6]. More recently, the two zoonotic species *Plasmodium knowlesi* and *Plasmodium simium* have also been associated with human malaria cases. The former usually infects macaque monkeys in Southeast Asia [7, 8], while the latter infects howler and capuchin monkeys and, very recently, has caused an outbreak of human malaria in southern Brazil [9].

In non-endemic countries, the identification of donors at risk of *Plasmodium* infection has become an important issue especially with the increased travelling of people and migratory phenomena, leading to the implementation of blood safety policies [10]. To avoid the risk of TTM, some countries (namely USA and Canada) have adopted a deferral policy, which, however, might also be associated with the loss of blood supply [5, 11]. In the effort to guarantee blood safety without affecting blood availability, most European countries have instead introduced blood screening policies in order to reduce the deferral period for those individuals who come from or visited malaria endemic areas and thus considered at risk, although there is a lack of consensus regarding the length of deferral of donors with positive screening test results across countries [5, 12]. In Italy the relevant regulation (no. 219, October 2005) has been recently amended (November 2015), and currently requires individuals considered at risk based on donor questionnaire to be tested for anti-*Plasmodium* antibodies and, in case of a positive result, to be excluded from blood donation for 3 years [13].

In principle, a blood screening test could be based either on direct or indirect methods. Detection of *Plasmodium* parasites on Giemsa-stained thick and thin blood smears by microscopic examination has been the gold standard for malaria diagnosis for over a century, allowing both the determination of species and that of parasite density. The theoretical limit of detection of microscopy is 5 parasites/µl but sensitivity varies according to the microscopist’s experience in the 5–100 parasites/µl range [14]. Rapid diagnostic tests based on antigen detection via immunochromatographic methods have been introduced in malaria endemic countries to improve access to diagnosis and limit treatment to confirmed cases where microscopy is not accessible, but show limited sensitivity at parasite densities lower than 100 parasites/µl [15]. Nowadays, more sensitive (0.1–2 parasites/µl) diagnostic tools based on nucleic acid amplification tests detecting malaria parasite genetic material are available and are especially useful in order to detect asymptomatic subjects carrying parasites at sub-microscopic densities [16].

Indirect methods for the detection of anti-*Plasmodium* antibodies include indirect immuno-fluorescence antibody test (IFAT), a well-established technique in several European countries [5] which was recently (2017) discontinued from production from the manufacturer Biomérieux, and enzyme-linked immuno-sorbent assay (ELISA), now very popular in most laboratories. Detection of *Plasmodium*-specific antibodies has virtually no place in diagnosis of clinically acute malaria cases nor infection, while is among the diagnostic criteria used to determine chronic forms of malaria such as hyper reactive malarial splenomegaly. Nonetheless, serological tests are recommended by the World Health Organization and the European Directorate for the Quality of Medicines for the screening of candidate blood donors considered at risk of carrying an asymptomatic *Plasmodium* infection [17, 18]. This choice is based on the supposedly higher sensitivity of serology, which lacks the detection limit of microscopy and other direct methods.

However, establishing sensitivity of serology is a challenging task, as the magnitude and longevity of Plasmodium-specific antibody responses vary according to several host (e.g. age, length of residence in endemic country), parasite (e.g. parasite, strain, antigen) and environmental (transmission level) factors [19, 20]. Such task is particularly challenging in the non-homogenous population of blood donors in non-endemic countries. Furthermore, an objective assessment of the sensitivity of the antibody detection techniques currently in use for blood safety purposes has been addressed by only few studies [20–22].

The objective of this observational study was to evaluate five commercial ELISA kits as a malaria screening tool of blood donors.

## Methods

### Study population

The retrospective study was carried out using archived serum samples from subjects recruited in two Italian laboratories. Serum samples (N = 64) obtained from 38 patients hospitalized between 2014 and 2016 at the Centre for Tropical Diseases (CTD) of the IRCCS Sacro Cuore-Don Calabria Hospital (Negrar, Verona, Italy) were used. All subjects were either diagnosed with malaria by microscopy or had a previous history of malaria infection. Samples, previously tested by IFAT, were chosen based on IFAT titre, in order to cover a range of IFAT titres spanning from 0 to 1:10,240 (N = 8 for each of titres 0, 20, 80, 160, 320, 640; N = 4 for each of titres 1280, 2560, 5120, 10,240). Samples with IFAT titre = 0 were obtained from patients with ascertained malaria infection at a very early stage of infection, when antibodies are not yet present. However, subsequent tests performed during hospitalization, showed a progressive increase in IFAT titre over time. Serum samples from malaria naïve blood donors (N = 8) and from malaria naïve patients with other parasitic infections (N = 15: n = 3 *Leishmania* spp., n = 4 *Trypanosoma cruzi*, n = 4 *Strongyloides stercoralis* and n = 4 *Schistosoma* spp.) were also collected at CTD and included as negative controls.

Serum samples from candidate blood donors referring to the transfusion centres in Massa Carrara, Livorno, Lucca, Pisa and Viareggio—identified by the questionnaire as at-risk for malaria infection—were collected at the Parasitology Section of the Microbiology Unit of Pisa University Hospital (AOUP, Tuscany, Italy) between February 2016 and February 2017, and screened with DRG ELISA (N = 496). The present study included N = 36 sera samples from screened candidate donors: all samples with a positive DRG result (n = 26), as well as random selection of samples with a negative DRG result (n = 10). All serum samples were stored at − 80 °C until use.

### Immuno-fluorescence antibody test (IFAT)

IFAT assay (Biomérieux Italia S.p.A.-Firenze) was performed according to the manufacturer’s instructions. The target antigen of the assay consists of *P. falciparum* parasites obtained from in vitro culture on human red cells and coated on slides. According to the manufacturer, the specificity and sensitivity of IFAT compared to another antibody detection method (i.e. electrosyneresis) or clinical symptoms are 98.74% (95% CI 95.43–99.66%) and 98.04% (95% CI 89.55–99.95%), respectively. The test enables the detection of *Plasmodium*-specific antibodies and samples are considered negative when IFAT titre = 0.

### Enzyme linked immuno-sorbent assay (ELISA)

Five commercial ELISA kits were employed to test serum samples: (1) Dia.Pro Malaria Ab (Diagnostics Probes—Srl, Italy); (2) DRG Malaria ELISA (DRG Diagnostics GmbH, Germany); (3) Novatec NovaLisa malaria (NovaTec Immundiagnostica GmbH, Germany); (4) Euroimmun Anti-Plasmodium ELISA (Euroimmun AG, Germany); (5) BioRad Malaria EIA Test (BioRad, CA, USA). All kits were obtained as a kind donation from their respective companies.

#### Dia.Pro malaria Ab

The kit is based on recombinant proteins representing immuno-dominant epitopes of *Plasmodium* spp. (without species indication) and detects anti-*Plasmodium* IgG, IgM or IgA. The detection is based on a biotin-streptavidin-HRP system: recombinant *Plasmodium* spp. proteins are used at the bottom of the plate, for antibody-capture, and in a biotinylated form for the identification of the antigen–antibody complex.

#### DRG malaria ELISA and Novatec NovaLisa malaria

Both these kits use recombinant antigens from *P. falciparum* and *P. vivax* to detect IgG and IgM antibodies. Antibodies against *P. malariae* and *P. ovale* are also detected due to antigenic similarity.

#### Euroimmun anti-*Plasmodium* ELISA

Among the tested kits, the Euroimmun is the only one based on a mix of antigens from the five *Plasmodium* spp. most frequently infecting humans. Consequently, it detects IgG against *P. falciparum*, *P. vivax*, *P. malariae, P. ovale* and *P. knowlesi.*

#### BioRad malaria EIA test

The test uses HRP-conjugated *Plasmodium* proteins for the detection of the antigen–antibody complex, consequently IgG, IgM and IgA anti *P. falciparum* and *P. vivax* can be detected. Antibodies against *P. malariae* and *P. ovale* can also be detected due to cross-reaction. Alternative names for this test can be found in the literature: Lab21 Healthcare or Lab21 Newmarket, depending on the distributor [21].

Internal positive and negative controls were provided in each kit and samples were manually processed according to the manufacturer’s instructions. The internal validity of ELISA results was evaluated by checking the OD values of the internal control samples against the validity ranges specified in each kit, by building a standard curve using serial dilutions of a pool of hyperimmune sera, and by estimating experimental precision and reproducibility on duplicate samples (Additional file 1). The cut-off values were calculated, indexes were computed (OD_sample_/OD_cutoff_ × 10), and samples were classified as negative, positive or “grey zone” according to manufacturer’s instructions. Samples with a result falling in the “grey zone” cannot be defined as positive or negative and, according to the kits instructions, the subject should be tested again after a few weeks.

### Study design and sample size

The sensitivity and specificity of the different ELISA kits was determined by testing sera (N = 64) from malaria patients and using IFAT as the reference standard. The specificity of the different ELISA kits was further examined by testing serum samples from malaria naïve individuals (N = 8 healthy subjects and N = 15 patients with other parasitic diseases). The agreement between ELISA and IFAT was further assessed by investigating the distribution of ELISA indexes and the proportion of positive ELISA results according to IFAT titre (0, 20, 80, 160, 320, 640, 1280 and 2560, 5120 and 10,240; 8 samples per group). In order to further compare ELISA kits with IFAT, and in particular to investigate whether a positive relationship exists, as expected, the association of IFAT titres with ELISA quantitative (indexes) and qualitative (positive/negative) results was assessed by graphical inspection.

The agreement between the five different ELISA kits was evaluated through the concordance between results of each pair of kits in samples from candidate blood donors (N = 36), in order to evaluate the probability that the same candidate donor will receive the same response independently of the kit used in the laboratory. The sample size was not formally calculated, as it was constrained by the number of tests supplied free of charge by the distributors.

### Statistical analysis

Statistical analyses were performed in STATA v.15 (StataCorp. 2017. *Stata Statistical Software: Release 15*. College Station, TX: StataCorp LLC) and GraphPad Prism v7.02 (GraphPad Software, Inc., CA, USA). The accuracy (sensitivity, specificity and the corresponding 95% CI) of the ELISA kits was computed to establish the ability of each test to classify samples (N = 64) as positive or negative, when compared to IFAT results. Samples falling in the “grey zone” were excluded from these calculations. Concordance and Cohen’s *k* statistics were computed to assess the agreement of qualitative results between each pair of the five ELISA kits in the sample of candidate blood donors. A p-value < 0.05 was considered statistically significant.

## Results

### Comparison of ELISA and IFAT results

**Fig. 1:**
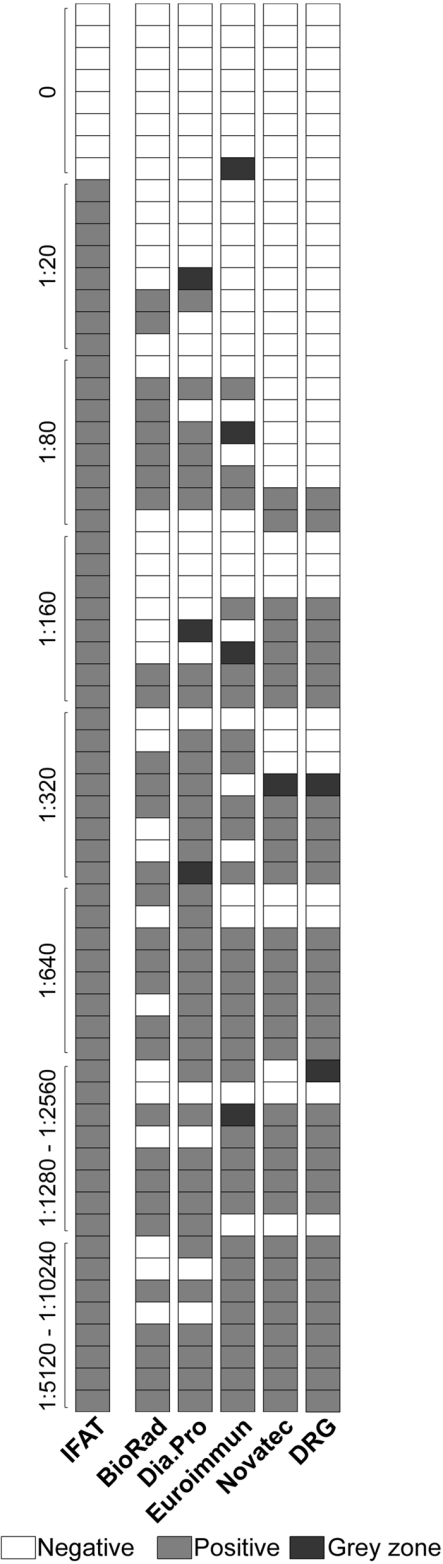
Qualitative comparison of the results obtained with ELISA and IFAT. The results obtained with the five ELISA kits and with the IFAT reference standard on N = 64 serum samples from malaria patients are reported. For IFAT, samples were classified as negative if IFAT titre = 0, or positive if IFAT titre ≥ 20; IFAT titres are reported on the left of the graph. For ELISA, samples were classified as negative, positive or “grey zone” according to the indications of the manufacturer of each kit

The ability of the five ELISA kits in classifying malaria serum samples as positive or negative for anti-*Plasmodium* antibodies, compared to the IFAT reference standard is reported in Table 1 and Fig. 1. Among the N = 64 tested samples, those falling in the “grey zone” were excluded from these calculations, since a positive or negative result could not be assigned. The number of samples in the “grey zone” was 3 for the Dia.Pro kit, 1 for Novatec, 4 for Euroimmun and 2 for DRG. No relation between these samples and the IFAT titre was observed.

**Table 1:** Performance of the five ELISA kits for the qualitative classification of malaria patients serum samples compared to IFAT

	BioRad	Dia.Pro	Euroimmun	Novatec	DRG
%SE (95% CI)	53.6 (39.7–67)	64.2 (49.8–76.9)	56.6 (42.3–70.2)	54.5 (40.6–68)	55.6 (41.4–69.1)
%SP (95% CI)	100 (63.1–100)	100 (63.1–100)	100 (59–100)	100 (63.1–100)	100 (63.1–100)

IFAT was considered as the reference standard: sensitivity was assessed in comparison to IFAT positive samples (N = 56) and specificity on IFAT negative samples (N = 8). Since samples with ELISA indexes falling in the “grey zone” were excluded from the analysis, the number of samples included for each test was as follows: BioRad n = 64; Dia.Pro n = 61; Euroimmun n = 60; Novatec n = 63; DRG n = 62

*SE* sensitivity, *SP* specificity, *CI* confidence interval

All kits showed 100% specificity (N = 8) and thus no false positive results. The sensitivity, instead, varied from 53.6% (BioRad kit) to 64.2% (Dia.Pro kit). Overall, the Dia.Pro showed the best performance, among the tested kits (Table 1).

All sera (N = 23) from healthy subjects with no history of malaria exposure (n = 8) as well as from patients suffering from other parasitic diseases (n = 15) resulted negative when tested with each of the five kits, confirming 100% specificity as well as lack of cross-reactivity with non-Plasmodium-specific antibodies.

**Fig. 2:**
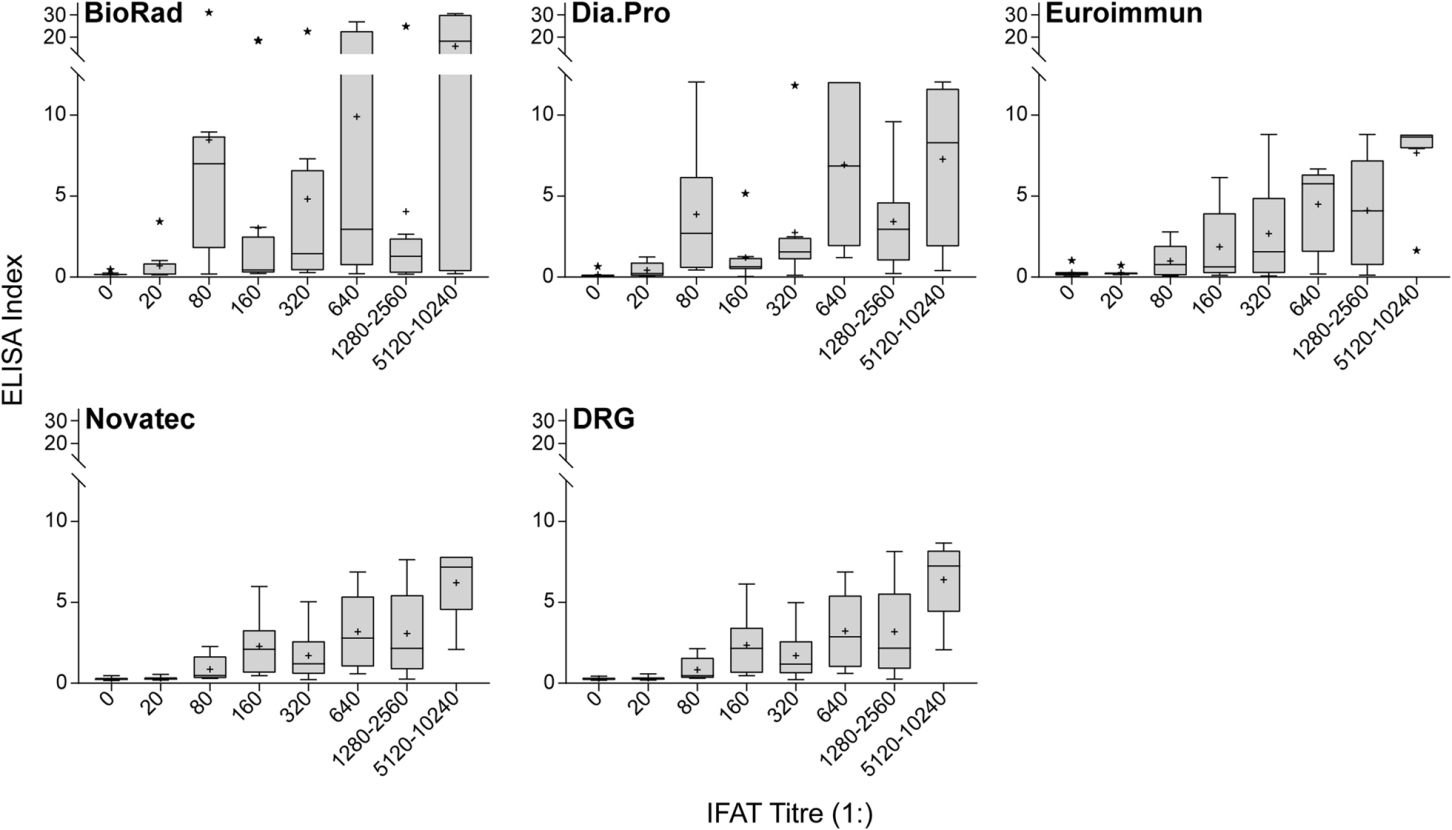
Distribution of ELISA indexes according to IFAT titres. The Figure shows, for each ELISA kit under study, a Tukey box-plot describing the distribution of ELISA indexes (OD_sample_/OD_cutoff_ × 10), according to IFAT titre. For each category represented, N = 8

Figure 2 shows the distribution of ELISA indexes according to IFAT titre for every ELISA kit. ELISA indexes increased at higher IFAT titres for the DRG, Euroimmun and Novatec kits, as expected. However, for the DRG and Novatec kits a distribution of ELISA indexes values lower than expected was observed at IFAT titre 1:320, while for the Euroimmun kit there was a wider distribution of ELISA indexes values in the IFAT titre range 1:1280–1:2560. Although for these three kits a positive linear relationship was observed between IFAT and ELISA measures, it was not possible to assign a given IFAT titre to a sample based on its ELISA index, due to some overlap in the indexes distribution. It was not possible to recognize an increase of ELISA indexes with IFAT titre and, therefore, a positive linear pattern, for the BioRad and Dia.Pro kits.

**Fig. 3:**
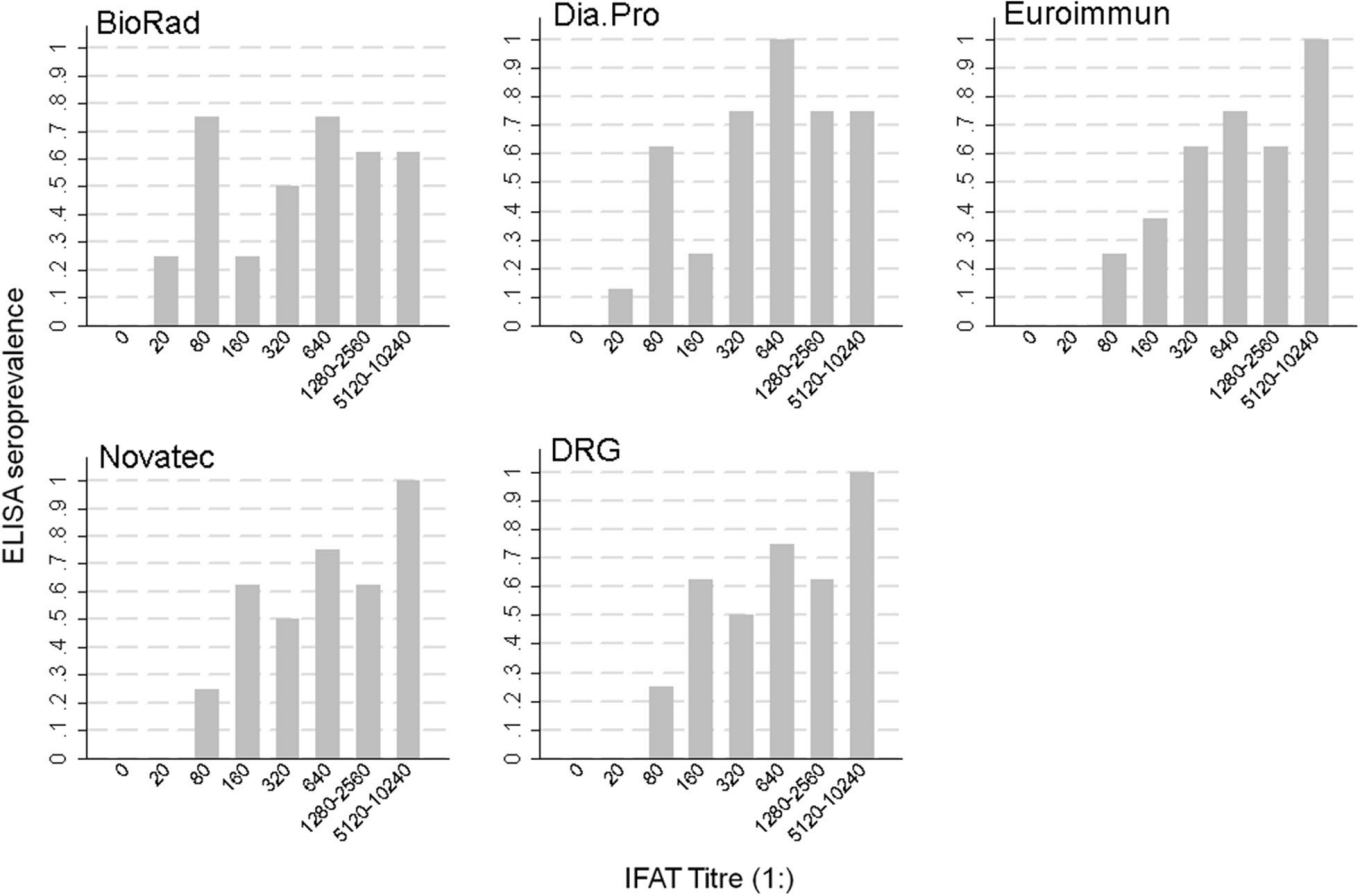
Frequency of ELISA positive results according to IFAT titre. The Figure shows, for each ELISA kit under study, the frequency of positive results according to IFAT titre. For each category represented, N = 8

Figure 3 shows the percentage of positive ELISA results according to IFAT titre for every ELISA kit. ELISA positivity (%) increased at higher IFAT titres for the DRG, Euroimmun and Novatec kits, as expected, with a minimum of 0% positivity at the lowest positive titre 1:20 and a maximum of 100% positivity at the highest titre 1:5120–1:10,240. However, for the DRG and Novatec kits a positivity lower than expected (i.e. no increase with respect to the previous titre) was observed at titres 1:320 and 1:1280–1:2560, while for the Euroimmun kit such deviation from expectation was observed at titre 1:1280–1:2560. For the BioRad and Dia.Pro kits, a minimum positivity was observed at the lowest titre, but at the other titres the pattern of positivity was not clearly linearly related to IFAT values.

### Comparison of ELISA kits for the screening of serum samples from candidate blood donors

The five ELISA kits were used to analyse serum samples from candidate blood donors and to compare their ability in classifying subjects as negative or positive at TTM screening. The concordance among the qualitative results of different ELISA kits is shown in Table 2. The paired kits showed variable agreement in the 41.7–94.4% range and *k* ranged from poor (0.1572, p = 0.0234) to very high (0.8750, p < 0.0001). Furthermore, only 12 out of 36 samples (33%) were identically classified as positive or negative by the five kits. The agreement between assays was not affected by the country of birth (malaria-endemic vs non-endemic) of candidate blood donors nor by the result (positive vs negative) of the original screening test (data not shown). Repeatability data for the DRG assay are presented in Additional file 1.

**Table 2:** Agreement between results of the five ELISA kits on sera from candidate blood donors

Pair of ELISA kits	% agreement	Cohen’s *k* (*p*-value)
BioRad vs. Dia.Pro	94.4	0.875
		(< 0.0001)
BioRad vs. Euroimmun	88.9	0.7647
		(< 0.0001)
Dia.Pro vs. Euroimmun	86.1	0.7073
		(< 0.0001)
BioRad vs. DRG	77.1	0.4928
		− 0.0018
Dia.Pro vs. DRG	74.3	0.4395
		− 0.0022
DRG vs. Euroimmun	68.8	0.3905
		− 0.0078
Euroimmun vs. Novatec	50.0	0.2202
		− 0.0078
DRG vs. Novatec	48.6	0.2115
		− 0.0098
BioRad vs. Novatec	47.2	0.1972
		− 0.0108
Dia.Pro vs. Novatec	41.7	0.1572
		− 0.0234

## Discussion

Serological methods are currently employed in Italy and other non-endemic countries for the screening of candidate blood donors in order to detect potential asymptomatic carriers of *Plasmodium* parasites and prevent transfusion-transmitted malaria [2, 5]. According to the National regulation, Italian laboratories can adopt different serological assays for the detection of *Plasmodium*-specific antibodies [13]. The aim of the present study was to evaluate and compare five commercial ELISA kits for the detection of anti-*Plasmodium* antibodies in both malaria patients and candidate blood donors.

Overall, the five ELISA kits showed unsatisfactory sensitivity (below 65%) for the classification of malaria samples, as compared to the Biomérieux IFAT, as reference test. Although no cross-reactivity with other parasitic infections was observed, such a low sensitivity indicates that these kits are inadequate to achieve an accurate detection of samples positive for the presence of anti-*Plasmodium* antibodies, due to the high proportion of false negative results [22–27].

The sensitivity of ELISA assays compared to IFAT among malaria patients’ samples has been previously assessed by three studies, all evaluating the Cellabs ELISA kit [22–24]. In the larger and most recent study (n = 144), conducted by Silvie and colleagues, a similar sensitivity to the present study was found (57%) compared to an in-house IFAT method, and the authors observed that ELISA sensitivity increased at higher IFAT titres [24]. In the first study by Chiodini and colleagues (n = 56), a very high sensitivity (93%) had been observed compared to an in-house IFAT method [22], but this result was not replicated in the following study by Mertens and colleagues (n = 49), where a higher although insufficient sensitivity (71%) was observed compared to Biomerieux IFAT [23]. A further evidence that ELISA sensitivity is insufficient to identify at risk donors is provided by a case of TTM occurred in 2015 in Georgia, USA, where the donor had a negative ELISA result [28].

When assessed on candidate blood donors’ samples, the five kits showed highly variable results, with a percentage agreement varying from poor to high, depending on the pair of kits. It is therefore possible that a candidate blood donor will receive a different response from laboratories using different ELISA kits, with a probability depending on the kits pair, implying that a candidate blood donor could be considered suitable for or excluded from donation, depending on the kit in use in the reference laboratory.

The present study is based on a limited sample size, which affects the precision of the estimates, and limits the generalizability of the results. Also, only ELISA kits commercially available in Italy at the time of the study were evaluated, and other products might show different characteristics. However the data presented here should provide evidence that the tested commercial ELISA kits are unsuitable—in particular if employed individually—for the screening of candidate blood donors for TTM risk.

## Conclusion

Semi-immune individuals represent the biggest challenge for blood safety and TTM screening as they might become asymptomatic carriers with a very low parasite density, which is difficult to detect with current direct diagnostic methods. Seed and colleagues reported that parasite densities as low as 1–10 parasites per blood unit are estimated to be sufficient to generate a malaria infection in a naïve recipient, albeit such levels are far from being detected by current methods, including PCR [6, 29]. Therefore, serological examination, combined with donors’ questionnaire, has been considered the most effective and sensitive screening method in non-endemic countries, although not ideal [6, 30].

In agreement with the literature, the present results highlight the need for the development of more sensitive serological tests, which should also be highly specific and reproducible. Moreover, future revisions, or application guidelines, of the current recommendations should include specific indication regarding which ELISA kit(s) should be adopted by the laboratories for TTM risk screening, in order for serology results and donors screening to be uniform and reproducible at national level.

To ensure blood safety and blood supply, the screening strategy may benefit from the use of multiple tests including more sensitive serological tests and molecular methods for the detection of *Plasmodium* spp. nucleic acids [31]. With the objective of establishing an accurate method for blood testing, Kitchen and colleagues have recently tested a screening algorithm which combines the use of different screening and confirmatory tests to detect parasitaemic blood donors, suggesting that a combination of multiple tests might be required to ensure safety of blood transfusions [1]. Such an approach would contribute to guarantee a safe blood supply avoiding the exclusion of suitable blood donors and, consequently, the loss of donations of rare blood.

## Additional file

**Additional file 1.** Internal validity of ELISA kits. Agreement between results of first and second DRG tests.

## Abbreviations

CI: confidence interval
ELISA: enzyme linked immuno-sorbent assay
HRP: horse radish peroxidase
IFAT: immuno-fluorescence antibody assay
OD: optical density
SE: sensitivity
SP: specificity
SSA: Sub-Saharan Africa
TTM: transfusion transmitted malaria
USA: United States of America
