# Supplementary material for: Risk of transfusion-transmitted malaria: evaluation of commercial ELISA kits for the detection of anti-Plasmodium antibodies in candidate blood donors
Source: Malar J. 2019 Jan 22;18:17. doi: 10.1186/s12936-019-2650-0 (PMC6341736; doi:10.1186/s12936-019-2650-0)
Supplement: Supplementary file 1 — Additional file 1. Internal validity of ELISA kits. Agreement between results of first and second DRG tests. [file 12936_2019_2650_MOESM1_ESM.doc]

**Risk of transfusion-transmitted malaria: evaluation of commercial ELISA kits for the detection of anti-*Plasmodium* antibodies in candidate blood donors**

Valentina Mangano ^1,2*^, Francesca Perandin ^3*^, Natalia Tiberti ^3^, Massimo Guerriero ^4^, Franca Migliaccio ^2^, Marco Prato ^2^, Lucia Bargagna ^2^, Stefano Tais ^3^, Monica Degani ^3^, Federica Verra ^3^, Zeno Bisoffi ^3,6@^, Fabrizio Bruschi ^1,5@^

**ADDITIONAL FILE 1**

**Internal validity of ELISA kits**

The OD values of the internal control samples (positive, negative, cut-off) lied within the validity ranges specified in each kit.

A standard curve was built using 10 serial dilutions of a pool of 3 hyperimmune (IFAT titre=10240) sera samples, and tested with each ELISA kit, showing the expected pattern of OD values.

Experimental precision was evaluated by testing a subset of samples (N=20) in duplicate within the same ELISA plate at the AOUP laboratory, calculating the Coefficient of Variation (CV) between indexes, and the agreement of qualitative results (positive/negative). The mean CV varied from a minimum of 8% for Novatec and Euroimmun to a maximum of 18% for Dia.Pro. Such low variability between indexes did not affect ELISA qualitative results that showed a 100% concordance between duplicate samples for all kits but Dia.Pro (94.5%).

Similarly, inter-laboratory reproducibility was evaluated by testing a subset of samples (N=10) in the two laboratories at AOUP and CTD. The mean CV varied from a minimum of 33% for Euroimmun to a maximum of 53% for Novatec. Such significant variability between indexes affected qualitative ELISA results that showed a concordance between duplicate samples in the 75-80% range, with the exception of the Euroimmun kit showing 100% concordance.

**Agreement between results of first and second DRG tests**

The agreement between the results of the first DRG test (DRG1) performed for the screening of candidate blood donors and of the second DRG test (DRG2) performed towards the objectives of the present study – a measure of repeatability of this assay – was 90% (9/10) for samples with a negative DRG1 result, and 46% (11/26) for samples with a positive DRG2 result, with an overall agreement equal to 55% (20/36).
